# Purification and Biochemical Characterization of a Specific β-Glucosidase from the Digestive Fluid of Larvae of the Palm Weevil, *Rhynchophorus palmarum*

**DOI:** 10.1673/031.009.0401

**Published:** 2009-03-12

**Authors:** Désiré Yapi Assoi Yapi, Dago Gnakri, Sebastien Lamine Niamke, Lucien Patrice Kouame

**Affiliations:** ^1^ Laboratoire de Biochimie et Technologie des Aliments de l'Université d'Abobo-Adjamé (Abidjan, Côte d'Ivoire), 02 BP 801 Abidjan 02, Côte d'Ivoire; ^2^ Laboratoire de Nutrition et de Sécurité Alimentaire de l'Université d'Abobo-Adjamé (Abidjan, Côte d'Ivoire), 02 BP 801 Abidjan 02, Côte d'Ivoire; ^3^ Laboratoire de Biotechnologie de l'Université de Cocody (Abidjan, Côte d'Ivoire), 22 BP 582 Abidjan 22, Côte d'Ivoire

**Keywords:** Coleoptera, Curculionidae, physicochemical characteristics, physiological role, transglucosylation

## Abstract

A β-glucosidase was purified from the digestive fluid of the palm weevil *Rhynchophorus palmarum* L. (Coleoptera: Curculionidae) by chromatography on anion-exchange, gel filtration, and hydrophobic interaction columns. The preparation was shown to be homogeneous on polyacrylamide gels, β-glucosidase is a monomeric protein with a molecular weight of 58 kDa based on its mobility in SDS-PAGE and 60 kDa based on gel filtration. Maximal β-glucosidase activity occurred at 55°C and pH 5.0. The purified β-glucosidase was stable at 37°C and its pH stability was in the range of 5.0–6.0. The enzyme readily hydrolyzed *p*-nitrophenyl-β-D-glucoside, cellobiose, cellodextrins and required strictly β-gluco configuration for activity. It cleaved glucose-glucose beta-(1–4) linkages better than β-(1–2), β-(1–3) and β-(1–6) linkages. The catalytic efficiency (K_cat_/K_M_) values for *p*-nitrophenyl-β-D-glucoside and cellobiose were respectively 240.48 mM^-1^s^-1^ and 134.80 mM^-1^s^-1^. Beta-glucosidase was capable of catalysing transglucosylation reactions. The yield of glucosylation of 2-phenylethanol (20 %), catalysed by the beta-glucosidase in the presence of cellobiose as glucosyl donor, is lower than those reported previously with conventional sources of beta-glucosidases. In addition, the optimum pH is different for the hydrolysis (pH 5.0) and transglucosylation reactions (pH 6.6).

## Résumé

Une β-glucosidase du suc digestif du charançon de palmier R. palmarum a été purifiée à homogénéité électrophorétique sur gel de polyacrylamide grâce aux chromatographies d'échanges d'ions, de gel filtration et d'interaction hydrophobe. La beta-glucosidase est une protéine monomérique avec un poids moléculaire de 58 kDa en électrophorèse sur gel de polyacrylamide en présence de SDS et de 60 kDa en gel filtration. Les températures et pH optima d'hydrolyse sont respectivement de 55°C et 5,0. L'enzyme reste stable à 37°C et aux pH compris entre 5,0 et 6,0. La beta-glucosidase hydrolyse fortement le p-nitrophényl-β-D-glucoside, le cellobiose et les cellodextrines. Elle est très spécifique de l'anomérie βet du résidu glucosyle. Elle hydrolyse mieux la liaison beta-(1–4) glucoside que les liaisons beta-(1–2) glucoside, β-(1–3) glucoside et β-(1–6) glucoside. Les efficacités catalytiques (Kcat/KM) afférentes à l'hydrolyse du p-nitrophényl-β-D-glucoside et du cellobiose sont respectivement de 240,48 mM-1s-1 et de 134,80 mM-1s-1. La β-glucosidase est capable de catalyser les réactions de transglucosylation. Le pourcentage de transglucosylation du 2-phényléthanol en présence du cellobiose comme donneur de glucosyle est faible par rapport à ceux obtenus avec les sources conventionnelles de β-glucosidase. Le pH optimum de transglucosylation (pH 6,6) est différent de celui de l'hydrolyse (pH 5,0). Une β-glucosidase du suc digestif du charançon de palmier *R. palmarum* a été purifiée à homogénéité électrophorétique sur gel de polyacrylamide grâce aux chromatographies d'échanges d'ions, de gel filtration et d'interaction hydrophobe. La beta-glucosidase est une protéine monomérique avec un poids moléculaire de 58 kDa en électrophorèse sur gel de polyacrylamide en présence de SDS et de 60 kDa en gel filtration. Les températures et pH optima d'hydrolyse sont respectivement de 55°C et 5,0. L'enzyme reste stable à 37°C et aux pH compris entre 5,0 et 6,0. La beta-glucosidase hydrolyse fortement le *p*-nitrophényl-β-D-glucoside, le cellobiose et les cellodextrines. Elle est très spécifique de l'anomérie βet du résidu glucosyle. Elle hydrolyse mieux la liaison beta-(1–4) glucoside que les liaisons beta-(1–2) glucoside, β-(1–3) glucoside et β-(1–6) glucoside. Les efficacités catalytiques (K_cat_/K_M_) afférentes à l'hydrolyse du *p*-nitrophényl-β-D-glucoside et du cellobiose sont respectivement de 240,48 mM^-1^s^-1^ et de 134,80 mM^-1^s^-1^. La β-glucosidase est capable de catalyser les réactions de transglucosylation. Le pourcentage de transglucosylation du 2-phényléthanol en présence du cellobiose comme donneur de glucosyle est faible par rapport à ceux obtenus avec les sources conventionnelles de β-glucosidase. Le pH optimum de transglucosylation (pH 6,6) est différent de celui de l'hydrolyse (pH 5,0).

## Introduction

Cellulose, the major component of plant cell walls, is the most abundant polysaccharide in nature and a virtually inexhaustible source of renewable bioenergy (Priit et al. 2001). Its hydrolysis primarily depends on at least three enzymes. These include several endo- and exo-cellulases and β-glucosidase or cellobiase. The former two enzymes can degrade native cellulose synergistically to generate cellobiose that is a product inhibitor for these enzymes (Bhat and Bhat 1997). Cellobiase plays an important role of scavenging the end product cellobiose by cleaving the β (1–4) linkage to generate D-glucose and also in the regulation of exo- and endo-cellulase synthesis. Furthermore, when a β-glucosidase preparation is added to lignocellulosic materials, it plays a major role in release of phenolic compounds, suggesting that cellulose degrading enzymes may also be involved to facilitate the breakdown of polymeric phenolic matrices (Zeng and Shetty 2000). This enzyme is widely spread in nature, predominantly being produced by microorganisms such as molds, fungi and bacteria (Bayer et al. 1998). β-glucosidases from fungi and bacteria have been studied extensively (Gueguen et al. 2001; Saha et al. 2002; Saloheimo et al. 2002; Li et al. 2002; Wallecha and Mishra 2003). However, little attention has been paid to β-glucosidases from insects. The only accounts in the literature refer to enzymes isolated from midgut cells of *Rhynchosciara americana* larvae (Ferreira and Terra 1983), midgut cells of the cassava hornworn *Ennnysis ello* (Santos and Terra 1985), the termites *Macrotermes mulleri* (Rouland et al. 1992), *Macrotermes bellicosus* (Matoub 1993) and *Macrotermes subhyalinus* (Kouamé et al. 2005a), tissues of the sugar cane borer *Diatraea saccaralis fabricius, Spodoptera frugiperda* (Marana et al. 2000), and the midgut of the yellow mealworm *Tenebrio molitor* larvae (Ferreira et al. 2002). Most of these enzymes were shown to have the polyspecificity between gluco-, fructo-, fuco-, galacto- xylo- and arabino-based substrates (Marana et al. 2000; Ferreira et al. 2002; Li et al. 2002; Wallecha and Mishra 2003; Kouamé et al. 2005a).

In this study, we attempted to purify and characterize the β-glucosidase from the digestive fluid of larvae of the palm weevil *Rhynchophorus palmarum* L. (Coleoptera: Curculionidae). This was done in order to find new β-glucosidase for use in glycobiotechnology and propose a biological role for the characterized enzyme in the degradation of cellulose.

## Materials and Methods

### Chemicals

Substrates: cellobiose, sucrose, sophorose, laminaribiose, gentiobiose, xylobiose, lactose, maltose, arabino-galactan, carboxymethylcellulose, inulin, laminarin, xylan, lichenan, starch, glucose, xylose, cellodextrins and *p*-nitrophenyl-glycopyranosides were purchased from Sigma Aldrich, www.sigmaaldrich.com. DEAE—Sepharose CL-6B, Sephacryl-S100 HR, Phenyl Sepharose CL-6B gels were obtained from Pharmacia-LKB Biotech, www.Pharmacia.com. The chemicals used for polyacrylamide gel electrophoresis (PAGE) were from Bio-Rad, www.bio-rad.com. All other chemicals and reagents were of analytical grade.

### Biological material

*R. palmarum* larvae were obtained from the commercial oil palm, *Elaeis guineensis* Jacq. (Arecales: Arecaceae), plantation near the Université d'Abobo-Adjamé (Abidjan, Côte d'Ivoire). They were collected directly from the palm weevil and dissected in the laboratory.

### Enzyme samples

*R. palmarum* larvae were rinsed in cold water and blotted with filter paper. Guts were dissected in cold 0.9% KCl (w/v) solution and digestive content was removed and stirred in the presence of 0.9% KCl (w/v) solution before centrifugation at 6000 × g for 30 min. The supernatant was then stirred with 100 mM acetate buffer pH 5.0 for 30 min. The homogenate was centrifuged at 10,000 × g for 30 min. The collected supernatant constitued the crude extract. After freezing at -180°C in liquid nitrogen, the crude extract was stored at -20°C.

### Enzyme assays

Under the standard test conditions, β-glucosidase activity was measured by the release of *p*-nitrophenol from the substrate *p*-nitrophenyl-β-D-glucopyranoside. An assay mixture (275 µl) consisting of a 100 mM acetate buffer (pH 5.0), 1.25 mM *p*-nitrophenyl-β-D-glucopyranoside and enzyme solution, was incubated at 37°C for 10 min. The control contained all reactants except the enzyme. Determination of other *p*-nitrophenylglycosidase activities was carried out under the same experimental conditions. The reaction was stopped by the addition of 1M sodium carbonate (2 ml), and absorbance of the reaction mixture was measured at 410 nm.

Oligo-saccharidase activity was determined by measuring the amount of glucose or xylose liberated from oligosaccharide by incubation at 37°C for 10 min in a 100 mM acetate (pH 5.0), containing 10 mM oligosaccharide. The amount of glucose was determined by the glucose oxidase-peroxidase method (Kunst et al. 1984) after heating the reaction mixture at 100°C for 5 min. The hydrolysis of xylobiose was assayed by high-performance liquid chromatography (HPLC) after heating the reaction mixture at 100°C for 5 min.

Polysaccharidase activity was assayed by the dinitrosalicylic acid procedure (Bernfeld 1955), using 1 % (w/v) polysaccharide (arabino-galactan, carboxymethylcellulose, inulin, lichenan, laminarin, xylan and starch) as substrate. The enzyme (100 µl) was incubated for 30 min at 37°C with 200 µl buffer (100 mM acetate, pH 5.0) and 100 µl polysaccharide. The reaction was stopped by addition of 300 µl dinitrosalicylic acid and heating in boiling water for 5 min. The absorbance was read at 540 nm after cooling on ice for 5 min.

One unit of enzyme activity was defined as the amount of enzyme capable of releasing one µmol of *p*-nitrophenol or glucose per min under the defined reaction conditions. Specific activity was expressed as units per mg of protein (U/mg of protein).

### Protein assays

Protein concentrations were determined by method of Lowry et al. (1951) using bovine serum albumin as a standard. Protein elution profiles from chromatographic columns were monitored by measuring fractional absorbance at 280 nm.

### Purification procedures

Five ml of crude extract was loaded onto a DEAE-Sepharose CL-6B (2.4 × 6.5 cm) that had been equilibrated previously with 100 mM acetate buffer pH 5.0. The unbound proteins were removed from the column by washing with four column volumes of the same buffer pH 5.0. Proteins were eluted using a stepwise gradient with 0.1; 0.2; 0.3; 0.4; 0.5 and 1 M KCl in 100 mM acetate buffer pH 5.0. Fractions (4 ml each) were collected at a flow rate of 80 ml/h and assayed for enzyme activity. The active fractions were pooled and saturated overnight by 80 % ammonium sulfate in a cold room. The precipitated pellet was then separated by centrifugation at 6000 × g for 30 min and dissolved in 1 ml of 100 mM acetate buffer pH 5.0. The enzyme solution was loaded directly into a Sephacryl S-100 HR column (1.6 × 64 cm) that was pre-equilibrated with the same buffer pH 5.0. Proteins were eluted at a flow rate of 15 ml/h using 100 mM acetate buffer pH 5.0. Fractions of 1 ml were collected and active fractions were pooled together. The pooled fraction from the previous step was saturated to a final concentration of 1.7 M ammonium sulfate and applied on a Phenyl-Sepharose CL-6B column (1.4 × 5 cm) previously equilibrated with 100 mM acetate buffer pH 5.0 containing 1.7 M ammonium sulfate. The column was washed with equilibration buffer and the proteins retained were then eluted using a stepwise gradient of 1, 2, 0.8, and 0.4, 0 M ammonium sulfate in 100 mM acetate buffer pH 5.0. Fractions of 1 ml were collected at a flow rate of 14 ml/h and active fractions were pooled together. The pooled fraction was dialysed against 100 mM acetate buffer pH 5.0 overnight in a cold room.

### Homogeneity and molecular weight determination

To check purity and determine molecular weight, the purified enzyme was analyzed using SDS-PAGE electrophoresis on a 10% separating gel and a 4% stacking gel (Hoefer mini-gel system; Hoefer Pharmacia Biotech, www.hoeferinc.com), according to the procedure of Laemmli (1970) at 10°C and constant current 20 mM. Proteins were stained with silver nitrate according to Blum et al. (1987). The sample was denatured by a 5 min treatment at 100°C. Electrophoretic buffers contained sodium dodecyl sulfate (SDS) and beta-mercaptoethanol.

The native molecular weight of the enzyme was determined using HPLC gel filtration chromatography. The TSK (Sigma-Adrich) column (2.5 cm × 52 cm; QC-PAK GFC 200) was equilibrated with 20 mM acetate buffer (pH 5.0) containing sodium azide 0.5 % (w/v) and calibrated with beta-amylase (200 kDa), alcohol dehydrogenase (150 kDa), bovine serum albumin (66 kDa), ovalbumin (48.8 kDa) and cytochrome C (12.4 kDa). Fractions of 0.5 ml were collected at a flow rate of 0.5 ml/min.

### Temperature and pH optima

The effect of pH on β-glucosidase activity was determined by measuring the hydrolysis of *p*-nitrophenyl-β-D-glucopyranoside in a series of buffers at various pH values ranging from pH 3.6 to 8.0. The buffers used were acetate buffer (100 mM) from pH 3.6 to 5.6 and phosphate buffer (100 mM) from pH 5.6 to 8.0. The pH values of each buffer were determined at 37°C. β-glucosidase activity was measured at 37°C under the standard test conditions. The effect of temperature on β-glucosidase activity was followed in 100 mM acetate buffer pH 5.0 over a temperature range of 30 to 80°C using 1.25 mM *p*-nitrophenyl-β-D-glucopyranoside under the standard test conditions.

### pH and temperature stabilities

The stability of β-glucosidase was followed over the pH range of 3.6 to 8.0 in 100 mM buffers. The buffers were the same as those used in the study of the pH and temperature optima. After 2 h incubation at 37°C, aliquots were taken and immediately assayed for residual beta-glucosidase activity. The thermal stability of the enzyme was determined at 37 and 55°C after exposure to each temperature for a period from 10 to 140 min. The enzyme was incubated in 100 mM acetate buffer pH 5.0. Aliquots were drawn at intervals and immediately cooled in ice-cold water. Residual activities, determined in both cases at 37°C under the standard test conditions, are expressed as percentage activity of zero-time control of untreated enzyme.

### Determination of kinetic parameters

The kinetic parameters (K_M_, V_max_ and k_cat_/K_M_) were determined in 100 mM acetate buffer (pH 5.0) at 37°C. Hydrolysis of *P*-nitrophenyl-β-D-glucopyranoside was quantified on the basis of released *p*-nitrophenol as in the standard enzyme assay. Cellobiose hydrolysis was quantified by determination of released glucose, determined with oxidase-peroxidase method (Kunst et al. 1984) after heating the reaction mixture at 100°C for 5 min. K_M_ and V_max_ were determined from Lineweaver-Burk plot using different concentrations of *p*-nitrophenyl-beta-D-glucopyranoside (1–10 mM) and cellobiose (1–20 mM).

### Effect of chemical agents

The enzyme was incubated with 1 mM or 1 % (w/v) of different chemical agents for 20 min at 37°C (various cations in the form of chlorides). After incubation, the residual activity was determined by the standard enzyme assay using *p*-nitrophenyl-β-D-glucopyranoside as a substrate. The activity of enzyme assayed in the absence of the chemical agents was taken as 100%.

### Transglucosylatlon reaction

The ability of β-glucosidase from digestive juice of the palm weevil *R. palmarum* larvae to catalyse transglucosylation reactions was tested with cellobiose as glucosyl donor and 2-phenylethanol as the glucosyl acceptor. In a typical experiment, the transglucosylation reaction was carried out at 37°C in 2 ml 100 mM acetate buffer or 100 mM phosphate buffer containing an appropriate amount of β-glucosidase corresponding to 15 units of the considered enzymatic activity and various concentrations of glucosyl donor (cellobiose) and of glucosyl acceptor (2-phenylethanol) (Kouamé et al. 2001, 2005a, 2005b). The progress of the reaction was monitored at different times (between 1 and 36 h) by withdrawing aliquots (100 µl) which were heated at 100°C for 5 min. After filtration through a 0.45 µm hydrophilic Durapore membrane (Millipore, www.waters.com), the reaction mixture (20 µl) was analysed quantitatively by HPLC at room temperature. Chromatographic separation of sugars (cellobiose) were performed on a Supelcosyl LC-NH_2_ (5 µm) column (0.46 × 25 cm) from Supelco (www.sigmaaldrich.com) using acetonitrile/water (75:25 ; v/v) as the eluent, and monitored by refractometric detection. The flow rate was maintained at 0.75 ml min^-1^. 2-phenylethanol (PE) and phenylethylglucoside (PEGlc) were analysed on a Thermo Hypersil (www.thermo.com) (5 µm) column (0.46 × 25 cm) using methanol/water (35 : 65, v/v) as the eluent. Chromatographic separations were monitored at 257 nm using a constant flow rate of 0.45 mlmin^-1^ (Kouamé et al. 2001, 2005a, 2005b).

The transglucosylation percentage (T) may be expressed as:

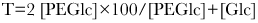

where [PEGlc] and [Glc] are concentrations of phenylethylglucoside and glucose respectively (Kouamé et al. 2001, 2005a, 2005b).

## Results

### Purification of beta-glucosidase

**Figure 1.**
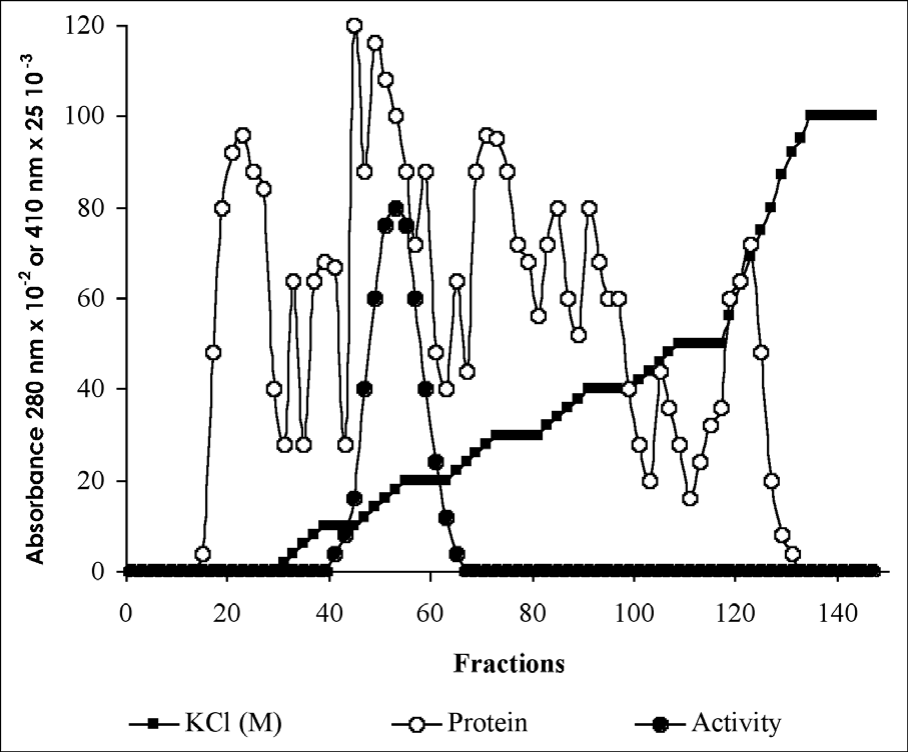
Anion-exchange chromatography of β-glucosidase from the digestive juice of the palm weevil *Rynchophorus palmarum* larvae on DEAE-Sepharose CL-6B.

**Figure 2.**
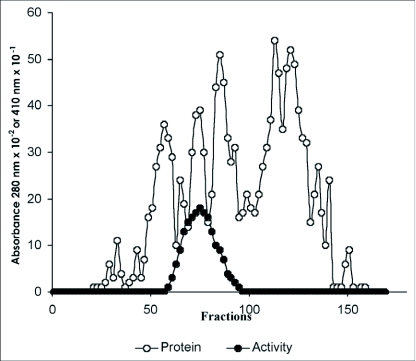
Gel-filtration chromatography of β-glucosidase from the digestive juice of the palm weevil *Rynchophorus palmarum* larvae on Sephacryl S100 HR.

**Figure 3.**
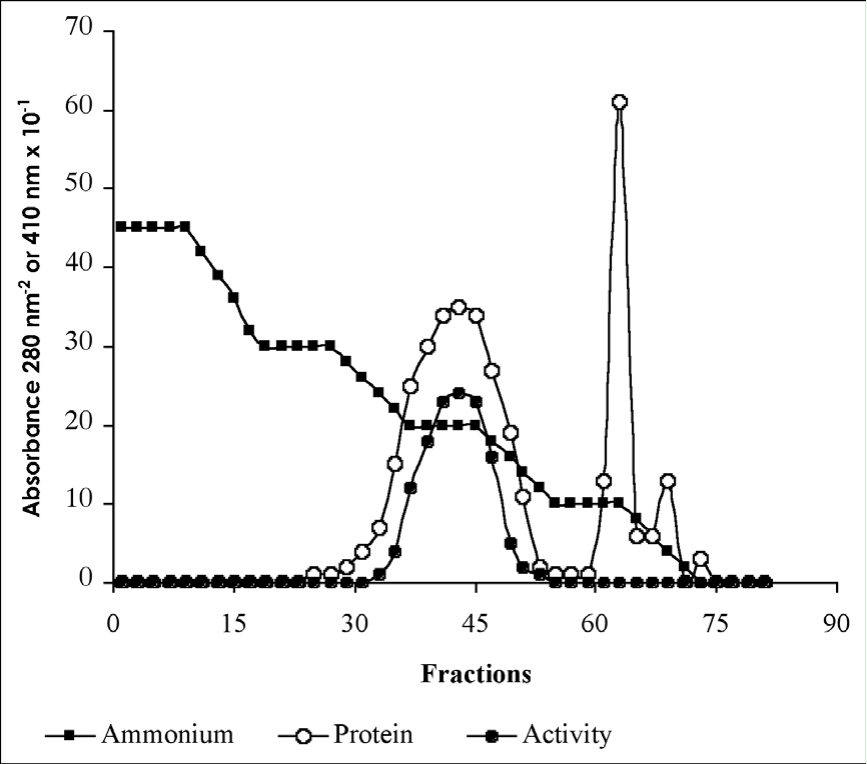
Hydrophobic interaction chromatography of β-glucosidase from the digestive juice of the palm weevil *Rynchophorus palmarum* larvae on Phenyl-Sepharose CL-6B.

**Figure 4.**
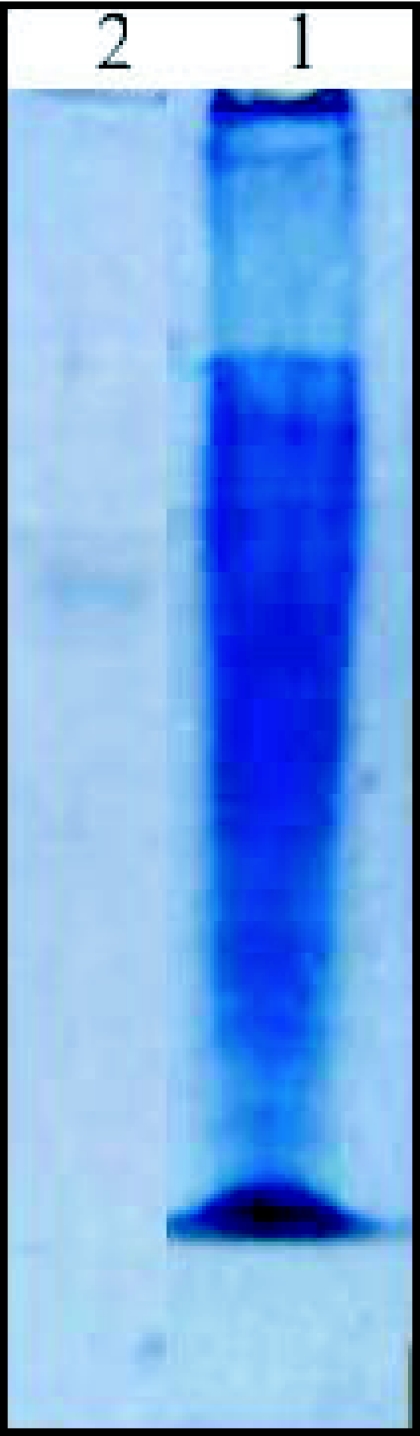
SDS-PAGE of purified β-glucosidase from the digestive juice of the palm weevil *Rhynchophorus palmarum* larvae. Sample was analysed in a 10% polyacrylamide gel. Lane 1, molecular weight markers (values in kDa); lane 2, purified beta-glucosidase.

A β-glucosidase was purified from the digestive juice of larvae of the palm weevil *R. palmarum. p*-nitrophenyl-β-D-glucopyranoside was used as the substrate to monitor enzymatic activity. Figures 1, 2 and 3 summarize the purification procedure, and Table 1 indicates the degree of purification and yield for each step. The purification protocol involves three steps of column chromatography (Figures 1, 2 and 3): anion-exchange, gel filtration and hydrophobic interaction. A single peak of activity was eluted at 0.2 M KCl from DEAE Sepharose CL-6B (Figure 1). Pooled fractions (47 to 59) showning β-glucosidase activity after this first step were subjected to gel filtration chromatography on Sephacryl S-100-HR. One peak showing β-glucosidase activity was resolved in this step (Figure 2). The pigments that are very abundant in the crude digestive juice, were almost completely removed during the gel filtration step. The β-glucosidase (pooled fractions 67 to 81) was further purified in a final step using hydrophobic interaction on phenyl sepharose CL-6B (Figure 3). The enzyme was eluted at 0.8 M ammonium sulfate. After purification, the β-glucosidase (pooled fractions 38 to 45) was enriched about 36.38—fold and the yield was 4.47 % (Table 1). The specific activity was 25.10 U/mg protein. The enzyme showed a single protein band on SDS-PAGE (Figure 4).

### Molecular weight

From the migration pattern of standards on SDS-PAGE, the molecular weight was calculated to be 58 kDa (Table 2). The molecular weight determined by HPLC was 60 kDa (Table 2).

### pH and temperature optima

**Figure 5.**
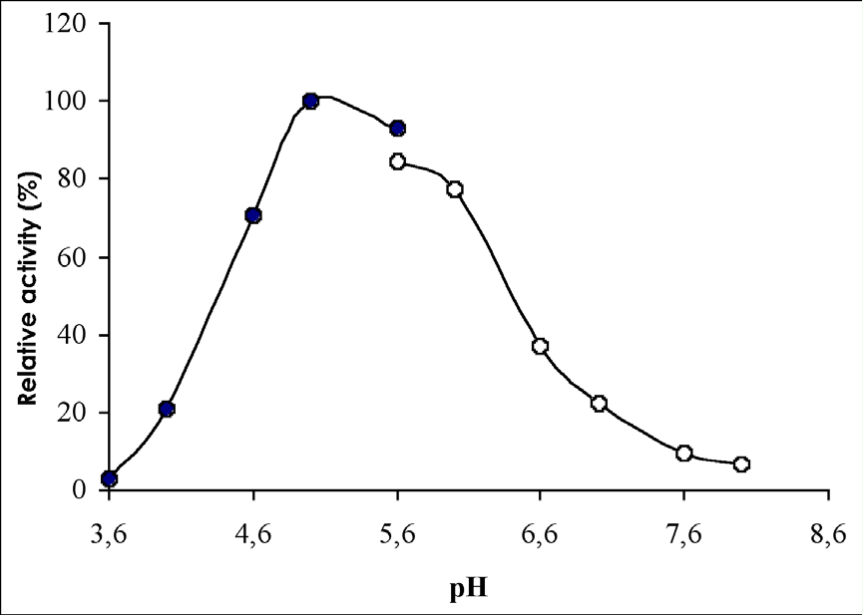
Effect of pH on the β-glucosidase activity from the digestive juice of the palm weevil *Rhynchophorus palmarum* larvae.

**Figure 6.**
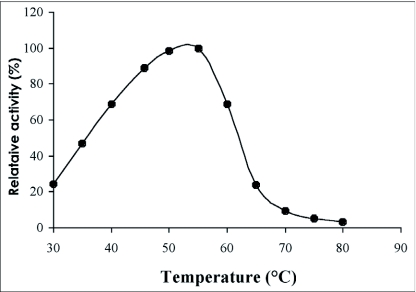
Effect of temperature on the β-glucosidase activity from the digestive juice of the palm weevil *Rhynchophorus palmarum* larvae.

The optimum pH for *p*-nitrophenyl-β-D-glucopyranoside hydrolysis was found to be 5.0. The enzyme retained more than 70% of its activity in the range pH 4.6 to 6.0 (Figure 5). The optimum temperature for the hydrolysis of *p*-nitrophenyl-β-D-glucopyranoside was 55°C (Figure 6) and the value of the temperature coefficient (Q_10_) calculated between 40°C and 50°C was 1.90 (Table 2). From the Arrhenius plot, the activation energy was found to be 68.78 KJ/mol (Table 2).

### pH and temperature stabilities

**Figure 7.**
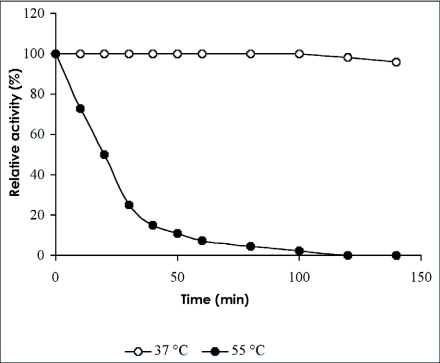
Thermal inactivation of the β-glucosidase from the digestive juice of the palm weevil *Rhynchophorus palmarum* larvae.

The enzyme was stable in the pH range of 5.0 to 6.0 for 120 min at 37°C (Table 2). It was unstable at 55°C, but at 37°C, the enzyme was stable for 100 min in 100 mM acetate buffer pH 5.0. At 55°C, the halflife of the β-glucosidase was 20 min and it completely lost its activity after treatment for 120 min (Figure 7).

### Substrate specificity and kinetic parameters

*R. palmarum* β-glucosidase did not attack the following *p-*nitrophenyl glycosides: α-glucoside, βand α-galactoside, αand β-mannoside, αand β-xyloside, αand β-L-arabinoside, βand α-fucoside, oligosaccharide: sucrose, lactose, xylobiose, maltose; or the polysaccharides arabinogalactan, carboxymethylcellulose, inulin, lichenan, laminarin, xylan and starch (Table 3). Although, the enzyme attacked sophorose and to lower degree laminaribiose and gentiobiose, it was clearly more active on cellobiose, cellodextrins (Table 3) and *p*-nitrophenyl-β-D-glucopyranoside.

The effect of substrate concentration on enzymatic activity was examined using cellobiose and *p*-nitrophenyl-β-D-glucopyranoside. With the two substrates, the enzyme obeyed the Michaelis-Menten equation (Table 2). The K_M_, k_cat_ and k_cat_/K_M_ values are reported in Table 4. The catalytic efficiency of beta-glucosidase, given by the k_cat_/K_M_ ratio is much higher for the *p*-nitrophenyl-β-D-glucopyranoside than the cellobiose.

**Table 1.**
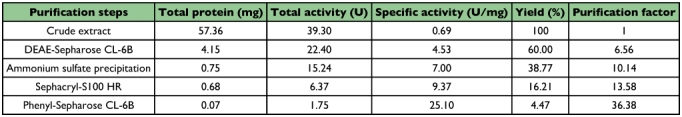
Purification of the β-glucosidase from the digestive juice of the palm weevil *Rhynchophorus palmarum* larvae.

**Table 2.**
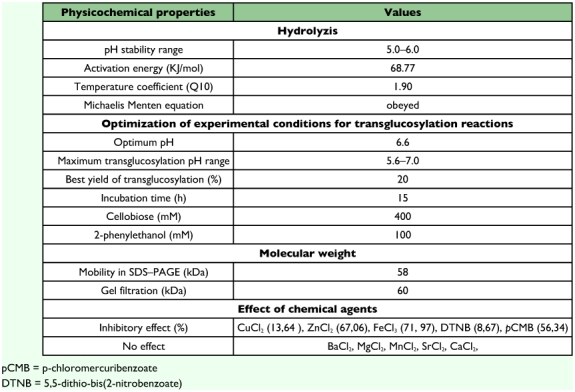
Some physicochemical characteristics of the bbeta-glucosidase from the digestive juice of the palm weevil *Rhynchophorus palmarum* larvae.

### Effect of chemical agents on enzyme activity

Chemical agents CuCl2 (13,64 %), ZnCl2 (67,06 %), FeCl3 (71,97 %), DTNB (5,5-dithio-bis(2-nitrobenzoate)) (8,67%) and *p*CMB (*p*-chloromercuribenzoate) (56,34 %) showed an inhibitory effect on β-glucosidase activity. Others had no effect on enzyme activity (Table 2).

### Transglucosylation reaction

**Figure 8.**
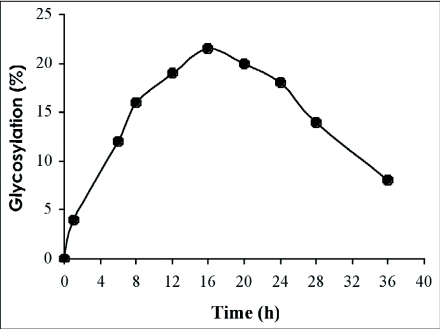
Time course of 2-phenylethylglycoside synthesis by the β-glucosidase from the digestive juice of the palm weevil *Rhynchophorus palmarum* larvae.

The ability of *R. palmarum* β-glucosidase f to catalyse transglucosylation reactions was tested with cellobiose as glucosyl donor and with 2-phenylethanol as glucosyl acceptor. Maximum transglucosylation was obtained in the range pH 5.6–7.0 (Table 2). A pH optimum of 6.6, different from that observed for the hydrolysis reaction, was obtained. Glucosylation kinetics were also studied as a function of the incubation time. In the initial stage of the reaction, considerable phenylethylglucoside was synthesised by the transglucosylation activity of the enzyme (Figure 8). However, as the reaction proceeded, the β-glucosidase hydrolysed the products formed. The efficiency of the *R. palmarum* β-glucosidase in catalysing of transglucosylation reactions was also largely dependent on the respective concentrations of the glucosyl donor, cellobiose and the glucosyl acceptor, 2-phenylethanol (Table 2). The best yields (20 %) were obtained with a concentration of about 400 mM of glucosyl donor (cellobiose) and 100 mM of glucosyl acceptor (2-phenylethanol).

## Discussion

A β-glucosidase was purified to homogeneity from the digestive juice of the palm weevil *R. palmarum* larvae. The purification protocol involves three steps of column chromatography: anion exchange, gel filtration, and hydrophobic interaction. Although DEAE-Sepharose chromatography led to a low purification factor, this step permitted the elimination of many pigments that are very abundant in the digestive juice. The specific activity of the β-glucosidase is lower than those obtained for the two β-glycosidases purified previously from *T. molitor* larvae midgut (Ferreira et al. 2001), the two β-glycosidases from the midgut of the sugarcane borer, *Diatraea saccharalis* (Azevedo et al. 2003) and the two β-glycosidases from worker of the termite *M. subhyalinus*. However, it is higher than those obtained for the β-glucosidases from worker of termite *M. mulleri* (Rouland 1992), *T. molitor* larvae lumen (Ferreira et al. 2003) and *Fusobacterium* K-60, a human intestinal anaerobic bacterium (Park et al. 2001).

The similarity in the molecular weights determined by denaturing SDS-PAGE and native gel filtration suggest that β-glucosidase is likely to be monomeric, as found in β-glucosidases from human liver (Mutoh et al. 1988) and *Thermus sp*.IB-21 (Kang et al. 2005).

The *R. palmarum* enzyme was inhibited by DTNB and *p*CMB. This suggests that a sulfhydryl group may be involved in the active site of the enzyme. This result is similar to that for the β-glucosidases from *Actinidia chinensis* (Ogawa 1990), *Leuconostoc mesenteroides* (Guegen *et al*. 1997) and *Tilapia* intestine (Taniguchi and Takano 2004) on beta-1,4 linkage.

A variety of glycosides were tested for their ability to serve as substrates. The *R. palmarum* β-glucosidase was inactive on high molecular mass polymer such as lichenan, xylan, carboxymethylcellulose, inulin, starch, arabinogalactan and laminarin. The purified enzyme had no contaminating glycosidase activities such as β-glucosidase, β-fucosidase, β-mannosidase, β-arabinosidase and β-xylosidase. The only substrates that were hydrolyzed by the enzyme were cellobiose, cellodextrins, laminaribiose, sophorose, gentiobiose and *p*-nitrophenyl-β-D-glucopyranoside. These results suggest that this β-glucosidase is an exo-glycosidase with a high specificity for the beta-anomeric configuration of the glucosidic linkage. This pattern is similar to the activity of the β-glucosidases from *Sclerotium rolfsii* (Shewale and Sadana 1981) and *Aspergillus niger* (Watanabe et al. 1992). The substrate specificity of the purified β-glucosidase differs from that found for most insect β-glucosidases. Its inability to cleave alphalinkages is commonly seen for purified β-glucosidases. This high substrate specificity suggests that the β-glucosidase could be used as a tool in the structural analyzis of D-glucose containing oligosaccharide chains of glycoproteins, glycolipids, and cellulose. The enzyme exhibited Michaelis Menten type kinetics and the KM for cellobiose (a key product of cellulose hydrolysis by exo- and endo-glucanase) is much lower than those reported for *Aspergillus* species (Workman and Day 1982; Wase et al. 1985). The low KM value obtained, compared with other organisms (Sternberg et al. 1977), suggests that this purified enzyme has high affinity towards its substrate and could possibly be used for industrial saccharification. The physiological role of β-glucosidase for *R. palmarum* larvae is the digestion of di and oligo-saccharides derived from cellulose.

Transglycosylation is a type of hydrolysis in which the glycosyl moiety of the substrate, instead of water, is transferred to other hydroxy compounds (Takegawa et al. 1998). Beta-glycosidases are now widely used to catalyse the synthesis of oligosaccharides and neoglycoconjugates by transglycosylation reactions. The regioselectivity of the formed compounds and yield depend on the enzymatic source, the structure of the glycosyl donor and acceptor, pH and temperature (Vetere and Paoletti 1996 ; Fourage and Colas 2001). This is why the ability of the β-glucosidase from *R. palmarum* to catalyse transglucosylation reations was tested with cellobiose as glucosyl donor and 2-phenylethanol as the glucosyl acceptor. The latter has already been used to study the transglycosylation activities of β-glycosidases from *Aspergillus oryzae* (Fortun and Colas 1991), *Achatina achatina* (Leparoux et al. 1997), *Thermus thermophilus* (Dion et al. 1999) and *M. subhyalinus* (Kouame et al. 2001, 2005a, 2005b) owing to easy and quantitative measurement by UV-absorbance of the tranglycosylation products, phenylethylglycosides. The experimental conditions were optimized in relation to those factors able to have an influence on the rate of transglycosylation. However, it must be noted that the β-glucosidase from the digestive juice of the palm weevil *R. palmarum* larvae operate at an optimum pH for the transglucosylation reaction at pH 6.6, which is different from that of the hydrolysis reaction (pH 5.0). To explain these differences, Huber et al. (1983) suggested that there is a group with a high pKa value at the active site that affects hydrolysis but not transglycosylation. A further finding was that the rate constant for the breakage of the glycosidic bond decreased with pH in a manner different from the change observed for the hydrolytic rate constant (Huber et al. 1983). In the case of *R. palmarum* larvae β-glucosidase, the difference of the two pH optima for hydrolysis and glucosylation is not significant and presents no easy opportunity for carrying out the synthesis reaction without rapid hydrolysis of the formed tranglucosylation products. The rate of hydrolysis is more highly favoured than the rate of transglucosylation product formation. The time course of the reaction is an important parameter since the products formed during the transglucosylation reaction are used as substrates by the enzyme, β-glucosidase from *R. palmarum* larvae hydrolysed the products formed. It seems that the product formed is the phenylethyl-β-D-glucoside. This result suggests that the β-glucosidase catalyses the splitting of β-glucosyl residue from non-reducing terminal of the substrate to liberate β-glucose. This behavior indicates that this enzyme operated by a mechanism involving the retention of the anomeric configuration, as is the case with glycosyl hydrolases belonging to families 1 and 3 (Henrissat 1991, 1998; Henrissat and Bairoch 1993). The glucosylation yields of 2-phenylethanol (20%) and catalysed by the purified β-glucosidase was much lower than those reported to date with other sources of β-glycosidases (e.g. *Escherichia coli, A. oryzae*).

The β-glucosidase from *R. palmarum* larvae appears to be distinct from other β-glucosidases so far reported in terms of substrate specificity and high affinity towards cellobiose. This enzyme could be used as a tool in the structural analysis of D-glucose containing oligosaccharide chains of glycoproteins, glycolipids and cellulose.

**Table 3.**
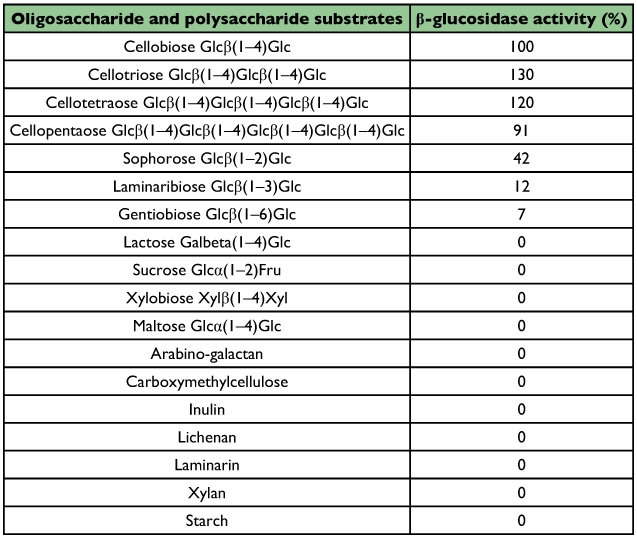
Activities of the β-glucosidase from the digestive juice of the palm weevil Rynchophorus palmarum larvae on oligosaccharide and polysaccharide substrates.

**Table 4.**
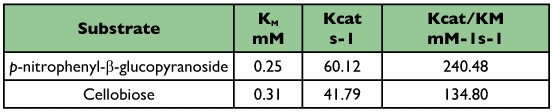
Kinetic parameters of the β-glucosidase from the digestive juice of the palm weevil *Rhynchophorus palmarum* larvae towards p-nitrophenyl-β-D-glucopyranoside and cellobiose.
